# Surgeons and preventative health: protocol for a mixed methods study of current practice, beliefs and attitudes influencing health promotion activities amongst public hospital surgeons

**DOI:** 10.1186/s12913-018-3606-8

**Published:** 2018-10-16

**Authors:** Stephen Barrett, Stephen Begg, Michael Kingsley

**Affiliations:** La Trobe Rural Health School, La Trobe University, PO Box 199, Bendigo, VIC 3552 Australia

**Keywords:** Surgeons, Health promotion, Professional practice, Hospitals, Attitude

## Abstract

**Background:**

The high prevalence of non-communicable diseases places significant demands on the healthcare system. As a result, hospitals are seeking to broaden their role to include more integrated health promotion. Strong leadership at different levels of the organisation is required for the successful integration of health promotion in hospital settings. The status of surgeons within healthcare affords them significant influence over clinical practice, and by extension, institutional policy and practice. The voice of this professional group is, however, absent from preventative health literature. The aim of this research is to identify which health promotion activities surgeons undertake, and to explore the attitudes of the profession towards health promotion activities.

**Methods:**

A mixed methods study will be conducted, guided by the principles of sequential explanatory design. Quantitative results from a clinician survey will be followed by in-depth, semi structured interviews to explore findings in more depth through qualitative analysis. We will recruit from general and orthopaedic surgeons and registrars in a major tertiary hospital in a regional city in Australia (n ≈ 31). Data will be collected, independently coded and analysed using a qualitative descriptive approach. Quantitative and qualitative results will be merged during interpretation to provide complementary perspectives of interrelated contextual factors that influence health promotion activities amongst hospital surgeons.

**Discussion:**

The depth of insight gained from these highly professionalised clinicians will offer a distinctive perspective on current practice, as well as the challenges of implementing effective health promotion into surgical practice. The findings from this research will assist in guiding strategy and policy at both clinical and institutional levels on health promotion planning and practice. Gaining insights from surgeons will strengthen the evidence base to assist the integration of health promotion into hospital practice.

**Electronic supplementary material:**

The online version of this article (10.1186/s12913-018-3606-8) contains supplementary material, which is available to authorized users.

## Background

Chronic non-communicable diseases are the leading cause of illness, disability and death worldwide [1]. These diseases include cardiovascular diseases, chronic respiratory diseases, diabetes, and some cancers [1], and are largely preventable [2]. There are a number of key reasons behind the rise in chronic diseases, including increasing life expectancy, the ageing of the population and behavioural health risk factors [1]. Chronic diseases have an intricate association with modifiable lifestyle risk factors, such as physical inactivity, poor nutrition, and smoking [3]. The increased prevalence of non-communicable diseases over the past number of decades has influenced the demands that are placed on the health system [4]. Hospitals, as a result, have been required to broaden their role from their primary focus of disease treatment towards a position of more integrated health promotion and holistic care [5].

Hospitals are in a suitable position within the health care system to be advocates for health promotion [6]. Hospitals represent the primary concentration of health resources, professional skills and medical technology in the community [5]. The extensive resources that hospitals command mean that even a small shift of focus has the potential to bring about an increase in resources dedicated to health promotion [5, 6]. This shift, over time, could bring potential health benefits to the community [5, 7]. The hospital healthcare system also provides important opportunities to reach the most disadvantaged in the community, who are often hard to reach by wider population-based approaches [8]. Moreover, hospital-based clinicians are seen as credible sources of advice and expertise on health issues that extend beyond their responsibilities for services related to sick care and can be strong advocates for health promotion [7].

Surgeons have an important role within the hospital system in the promotion of healthy lifestyles and behaviour change for patients with, or at risk of, chronic disease [9]. Due to their extensive training, specialisation and medical expertise, surgeons are perceived as dependable sources of advice and expertise on health issues that extend beyond their responsibilities for services related surgical care [6]. Surgeons as such, are influential in the promotion of lifestyle behaviour change [7]. The surgical profession is one of responsibility and leadership, playing key roles in strategic planning, especially pertaining to clinical care [10]. Strong leadership at different levels of the organisation has been indicated as the key element for integrating health promotion in a hospital setting [7, 8]. Hospital surgeons therefore have significant influence over clinical settings, and by extension, institutional policy and practice, necessary for the implementation of research into clinical practice [10].

The persuasive role of hospital doctors in promoting health-related behaviour change has been acknowledged [11]. There is however, a significant gap between potential and practice, with low rates of preventive health interventions reported by hospital doctors [12]. Only a small proportion of emergency physicians reported routine screening and counselling of patients about preventive health; furthermore, the majority were not confident in their ability to assist patients change their health-related behaviours [13]. When surveyed, hospital speciality clinicians also felt that they were not the most appropriate person to offer patients advice about behaviour change [12]. The authors suggested that this result provides an explanation for the low implementation rates of behaviour change interventions in the hospital setting [12]; however, no interviews were carried out to further probe the survey findings to gain a deeper understanding of the topic. Decisions on implementing evidence into surgical practice have been found to be multifactorial [14]. These decisions are influenced by local, contextual and social circumstances [15, 16], as well as organisational processes and available resources [15, 16]. Given the prevalence of chronic diseases and the recommendation for hospitals to broaden their role towards a position of more integrated health promotion [5, 7, 8, 17], it is important to gain insights from hospital surgeons due to the influence that they exert on patient behaviour and clinical policy and practice. Existing literature has reported qualitative and quantitative findings separately rather than linking quantitative data of actual clinical practice to qualitative data on beliefs and attitudes that influence such practices [18]. Therefore, a better understanding of how attitudes towards risk factor management influence clinical practice is required to guide future preventive health policy and practice.

The aim of this mixed-methods study is to identify which health promotion activities surgeons carry out in public hospitals, and to explore the attitudes of the profession towards health promotion practice. Combining quantitative data on levels of risk factor intervention with qualitative data about beliefs and attitudes will highlight ways in which interventions and practice can be implemented in clinical care to increase the overall rate of risk factor management. The depth of insight gained from the study of these highly professionalised clinical groups will offer a distinctive perspective on current practice, and the challenges of implementing effective health promotion into hospital settings. The results of this study have potential to guide health promotion policy and practice into the future.

## Methods/design

This study will use a mixed methods approach to explore which health promotion activities surgeons carry out in public hospitals, and to understand attitudes of surgeons towards health promotion activities. Quantitative data will be derived from a clinician survey. Semi-structured interviews will be used to obtain qualitative data. The mixed methods study will use a sequential explanatory design [19]. This is a two phase design where quantitative data are collected prior to the collection of qualitative data. The sequential explanatory design uses the qualitative results to further explain and interpret the findings from the quantitative component. The study framework is grounded in pragmatic epistemology [20, 21], to explore clinician views, behaviours and actions relating to preventive health practice. To promote transparency of our planned research, Table 1 presents qualitative research design aspects within the COnsolidated criteria for REporting Qualitative (COREQ) studies framework [22].

**Table 1:** Qualitative study design

Domain 1: Research team and reflexivity	
Personal characteristics	
1. Interviewer/facilitator	All interviews will be conducted by the same member of the study team.
2. Credentials	The interviewer will be a masters-level trained research assistant.
3. Occupation	The interviewer will be employed full-time as a project officer.
4. Gender	The interviewer will be male.
Relationship with participants	
6. Relationship established	Potential interviewees will be contacted with a standardised recruitment email to introduce the study and the interviewer and to request their participation.
7. Participant knowledge of the interviewer	The recruitment email will explain the study goals and why the interviewer is interested in conducting this research. This information will be reviewed at the start of each interview.
8. Interviewer characteristics	The recruitment email will provide information about the research team, including the interviewer. This information will be reviewed at the start of each interview.
Domain 2: Study design	
Theoretical framework	
9. Methodological orientation and theory	The qualitative portion of the study will use a qualitative description approach.
Participant selection	
10. Sampling	Potential interviewees will be selected based on their practicing status in the target hospital.
11. Method of approach	Potential interviewees will be approached with a standardised recruitment email.
12. Sample size	We anticipate conducting 5 to 10 interviews.
13. Non-participation	We will document any reasons provided by those who decline to participate as well as any individuals who do not respond to our recruitment email.
Setting	
14. Setting of data collection	Data will be collected via interviews conducted in person.
15. Presence of non-participants	We anticipate that the interviewer and interviewee will be the only individuals present.
16. Description of sample	The sample will include Orthopaedic and General surgeons and their registrars consulting out of the target hospital.
Data collection	
17. Interview guide	The interview guide will be developed by the study team. It will be pilot-tested and refined before data collection begins.
18. Repeat interviews	We do not anticipate conducting repeat interviews.
19. Audio/visual recording	Once permission is granted, interviews will be audio recorded.
20. Field notes	The interviewer will draft summary notes immediately after concluding each interview.
21. Duration	We anticipate that interviews will last no more than 30 min.
Domain 3: Analysis and findings	
Data analysis	
24. Number of data coders	We plan to have two coders pilot a sub-sample of transcripts. Once discrepancies are resolved and the codebook is finalised, the full set of transcripts will be coded by one individual.
25. Description of the coding tree	We plan to develop a coding tree (i.e., codebook) based on a review of the literature, a priori knowledge within the study team, and summary notes from interviews.
26. Derivation of themes	Themes will be derived once data have been coded. Preliminary themes may be identified based on discussions with the interviewer and review of field notes.
27. Software	We plan to use NVivo qualitative research software.
28. Participant checking	A bulleted list of key findings will be shared with participants once data have been coded and analysed.
Reporting	
29. Quotations presented	Quotations from interviews will be used to present findings, and they will be accompanied by an interviewee identification number.
30. Data and findings consistent	Our planned use of quotations will allow for assessment of consistency between our data and findings. We will also create supplemental tables with additional quotations to share as much information as possible when presenting our findings.
31. Clarity of major themes	We plan to use sub-headings listing our major themes to promote clarity when writing up our findings.
32. Clarify of minor themes	We plan to provide quotations from interviewees who raised minor themes or shared information contrary to findings of our major themes.

### Clinician survey

Surgeons will be invited to complete a short 17-item self-administered clinician survey to assess health behaviour risk factor management, perceived knowledge, confidence and attitudes towards preventative health (Additional file 1). The clinician survey asks respondents to report on proportions of clients seen over a recent period who they: (1) asked about smoking, nutrition, alcohol and physical activity; (2) assessed for readiness to change; (3) provided verbal and written advice on these risk factors and; (4) referred to other services for support in changing risk factors. These clinician practices are the dependent variables. The clinician survey also questions the participants about their knowledge and confidence in screening and managing each risk factor. In addition, attitudinal measures will be included for: (1) the clinicians’ opinion on the perceived effectiveness of the interventions; (2) the perceived importance of the intervention for the clients they see; (3) perceived work priority; and (4) perceived acceptability on the part of the client of raising the topic of lifestyle risk factors. The answers to these questions constitute the independent variables. All clinician survey items are measured on a 5-point Likert scale. The clinician survey has been adapted with permission from a previously developed instrument used to assess health behaviour risk factor management practices and capacity in community dwelling participants [18, 23].

### Semi- structured interviews

Qualitative data permits the recording of first-hand accounts of the individuals studied, offering rich, straight descriptions of experience or events [24, 25]. Following analysis of the clinician survey, semi-structured interviews will be conducted with a purposeful sample of surgeons and surgical registrars. Interviews will be structured in an attempt to gain participant’s opinions on factors that influence their decisions to undertake health promotion activities. Interviews offer value in understanding the perspective of individuals as well as the rationale for their actions [26]. The interviews will take place face-to-face where possible, with telephone interviews used where required for practical reasons.

This research will use qualitative description as the theoretical framework for the qualitative component [27]. Qualitative description provides straightforward, rich descriptions of experiences or events. Unlike other qualitative approaches which seek to develop new concepts or theories, the final outcome of qualitative description is a description of a participant’s’ experiences in a language similar to the participant’s own [27]. In qualitative description interviews are typically designed to focus on areas in health care that are either poorly understood and/or potentially amenable to intervention [27].

To facilitate uniformity, open-ended questions as well as question-related probes will be drafted. These questions will be designed to fit with the objectives of the research; however, they will not be finalised a priori, rather, they be will be built from the analysis of clinician survey answers [21]. Interview questions might be altered and adapted to differ between different groups of surgeons given the heterogeneity of the patients they see. Pilot interviews will be undertaken and audiotaped. The resulting interviews will be transcribed verbatim and used to refine the interview script. In keeping with the qualitative description framework, the interview script will be developed and amended formatively, based upon findings and emerging concepts [24, 28].

Descriptive information, such as years of practice and specialisation level, will be captured at the outset through the clinician survey. All participants will be encouraged to express opinions understanding that every answer is valuable and of use in the analysis [24, 29]. Full interviews will be audiotaped and transcribed verbatim. Field notes will be used to supplements audio and transcripts. After each interview the questions and answers will be assessed and reviewed to establish how well the interview script facilitated exploration of the topic. Where required, the script will be revised prior to the next interview [29].

### Ethics

The study has been approved by the Research Ethics Committees of the governing Hospital and University. Participants will choose to complete the clinician survey online or in hard copy. Participants who choose to complete the online version of the survey will be informed electronically, prior to commencing the survey that if they continue and complete the survey they agree to provide informed consent. Participants who choose to complete a hard copy version of the clinician survey will be given a Participant Information and Consent Form to complete prior to undertaking the clinician survey, as well as a stamped addressed envelope to return the consent form and clinician survey. Participants who choose to complete a hard copy version of the clinician survey will be reminded not to place their name on the clinician survey. Participants will be notified that their involvement is voluntary and can be withdrawn at any time, and that confidentiality is protected through the anonymization of all collected data. Any publication will not attribute specific comments to identifiable participants.

### Participants

The target participants for this study are general and orthopaedic surgeons, and their registrars who consult out of the elective outpatient clinic in a major tertiary hospital in a regional city in Australia. The clients presenting to this clinic are ambulatory, non-admitted clients. While this group may be seen as a representation of the general community-dwelling population, research indicates that patients presenting to hospital clinics are more likely to have higher rates of chronic disease than the general population [3, 4, 7].

As there are only a limited number of surgeons (general and orthopaedic) (*n* = 20) and registrars (*n* = 11) operating out of the study hospital, we will offer participation to all of these practitioners. Recruiting all surgeons and registrars consulting out of the hospital will increase the potential sample size, and provides a participant sample with differences in career stage and level of training. In qualitative description, the methodology seeks to gather enough data to saturate the explanation [27, 28]. Interviews will be continued until no interviewees are providing new information, and data saturation is determined [30]. If it is deemed that theoretical saturation is reached prior to full recruitment, then recruitment will discontinue [25, 27, 30].

We will recruit participants from one study site only. We have chosen to recruit participants from one study site only due to the heterogeneity that exists between hospitals in terms of infrastructure, clinical practice and resources, as well as broader contextual factors that may differ between areas serviced by hospitals such as the availability of, and access to, local preventative health providers and programs [31]. This study is part of a broader body of work, investigating health promotion in non-admitted secondary care patients. We have already undertaken a clinical trial investigating behaviour change interventions for non-admitted secondary patients in a single-hospital setting (ANZCTR trial id: ACTRN12616001331426). This proposed mixed-methods study aims to fill the gap in knowledge pertaining to surgeons’ actual involvement in the practice of health promotion in an ambulatory care setting; these results can be used in the design of subsequent clinical trials involving surgeons screening and recruiting secondary care patients for behaviour change interventions.

### Recruitment

An email will be sent to all potential participants by their head of department, explaining the rationale for the study and the inherent requirements. The email will explain that participants can take part in the clinician survey, the interview, or both. The email will contain a link to the electronic version of the clinician survey. The email will also inform participants that a hard copy version of the clinician survey is available if this is preferred. The email will contain contact details for the research team for those individuals who would like more information. To assist with completion rates [32], two subsequent reminder emails with attached clinician survey link will be sent out through the same electronic channel, at four-weeks and eight-weeks post initial email.

Individual participants will be approached to discuss involvement in the interviews, and where interested, a time will be arranged with each clinician to undertake the interview. Every effort will be made to allocate sufficient time in the interview to discuss informed consent and undertake the interview. Participants will be given a Participant Information and Consent Form to complete prior to undertaking the interview. Where appropriate, and to coordinate scheduling, the coordination of the interview times may be facilitated through the personal assistants of the surgeons. To facilitate the development of interview topics and question design, we aim to have at least 5 clinician surveys completed and analysed before undertaking any interviews.

### Analyses

#### Clinician survey

Consistent with published literature utilising this survey [18, 23], the relationship between the dependent variables (clinician practices) and independent variables (confidence, knowledge and attitudes) will be analysed. Due to the small sample of clinicians available for recruitment, dependant variables will be recorded as low, moderate or high. Low implementers will be defined as clinicians with screening and intervention scores, across all risk factors, in the first quartile. High implementers will be defined as clinicians with screening and intervention stores, across all risk factors in the fourth quartile. Given the relatively small sample size, a Generalized Estimating Equation will be used to estimate the parameters of a generalized linear model with a possible unknown correlation between outcomes [33]. The generalized estimating equation procedure extends the generalized linear model to allow for analysis of repeated measurements or other correlated observations [33]. Generalized estimating equations allows for the highlighting of moderators that do not directly correlate with the outcome of interest, but influence other related factors [33–35]. Analysis of the surveys will be used to offer exploratory themes, questions and question-related probes for the semi-structured interviews.

#### Interviews

Data from individual interviews will be collected and analysed promptly, to allow emerging themes to be added to and explored in following interviews. Qualitative description analysis requires the reading and re-reading of transcripts, allowing the development of a coding scheme that accurately reflects concepts in the text [25, 26]. Coding is a critical part of the data analysis stage and aims to merge concepts and themes that emanate from reviews of the interviews and corresponding text, taking the text from the descriptive to the interpretive [36]. The qualitative description process utilises open and axial coding of transcripts, which, in keeping with the concurrent methodological approach, will occur simultaneously [24]. Open coding describes the reading of interview transcription linearly with the aim of identifying concepts and then grouping concepts into categories and subcategories [36]. The analytic process may be used to identify the more general categories that these concepts are instances of, such as institutions, work activities, social relations and so forth [26]. Axial coding is the process of developing connections between code categories and sub-categories via a combination of inductive and deductive thinking [26]. Consistent themes are integrated, reducing the overall number of categories [26]. The emerging categories will be reviewed by the research team with the aim of using the categories to explain the factors that influence surgeons’ decisions to use health promotion activities.

Following open and axial coding, a process of selective coding is undertaken [37]. This process defines the central or core category, and its clear relationship to other categories [28, 37]. The core category has the analytic power to combine all categories to form an explanatory whole [28, 37]. Data will be coded and analysed independently by two investigators. The investigators will identify and code themes using NVivo 10.0 software (QSR International). An electronic codebook will be developed to assist with the coding scheme and data characterisation. The codebook will contain code definitions as well as rules related to each unique code. To improve reliability, a third investigator will review the codebook and samples of transcripts. Disagreement between investigators will be resolved through discussion, and where required, the thorough re-examining of transcripts. Categories will be represented visually using diagrams to illustrate the conceptual relationship between the emerging categories. See Table 1 for additional qualitative analysis details.

### Risk of Bias

This study seeks to avoid the weakness or intrinsic biases inherent in single method, single observer, and single theory studies by adopting a mixed methods approach [20]. It will involve a series of steps consistent with rigorous qualitative research [20, 21], including: note-taking during clinician interviews; systematic data coding and analysis; detailed documentation of analytic decisions to explicitly demonstrate the means of arriving at the codes, and to avoid overgeneralisation and speculative conclusions; including direct quotations from participants to offer readers some perspective on the evidence from which the study findings and conclusions are based; and reviewing data coding processes, analytic decisions, and resultant themes by the two investigators [38]. These steps allow for the triangulation of findings by the research team, with high degrees of team involvement required through the stages of data analysis and interpretation [28, 38]. This process is designed to increase rigor by decreasing the likelihood that substantial thematic ideas get overlooked, and ensure transparency in both data coordination and interpretation [28].

### Trial status

Further to the approval for the research from the ethics boards of the hospital and university, the study has also been approved by the Group Executive at the study hospital, including Executive Director of Acute Health and the Chief Medical Officer.

## Discussion

Hospitals play an important role in sick care, the provision of rehabilitation services, the promotion of health, and the prevention of disease [4]. These activities have been core components of hospital work; however, the increasing prevalence of lifestyle-related chronic diseases necessitates a more expanded scope, and standardised provision of initiatives to enable clients to take an active role in preventative health and chronic disease management [5, 7, 8]. This requires the reorientation of health care facilities to integrate health promotion, disease prevention and rehabilitation services in curative care [17]. Hospitals can have a strong influence on health behaviour, with patients demonstrating more responsiveness to health advice in situations when they are experiencing ill-health [39]. A fundamental necessity towards integrating health promotion in hospital settings is strong leadership at different levels of hospital governance [7, 8]. This leadership is epitomised by hospital surgeons, influential figures within both clinical settings, and by extension, hospital organisational culture and practice [16, 40]. Surgeons are seen as the predominant authoritative source of advice and expertise on health issues, having influential roles in both clinical and administrative structure [7, 10, 39]. In spite of this, information regarding the professional practice, and the opinions of these exemplars of clinical practice pertaining to preventative health are absent from the literature.

The primary outcomes of this study will be to: (1) identify what health promotion activities surgeons carry out in public hospitals, and (2) explore the attitudes of surgeons towards preventative health practice. The depth of insight gained from the study of these highly professionalised clinical groups will offer a distinctive perspective on current practice, as well as the challenges of implementing effective health promotion into surgical practice. The findings from this study will offer insight into individual, institutional and contextual factors that influence surgeons’ decisions to participate in health promotion activities.

There are a number of limitations to the proposed study design. A limited number of participants will be recruited for this mixed-methods study. The demographic characteristics of the surgeons who participate in this study will be captured to enable comparison to broader communities of surgeons. Additionally, participants will be recruited from one study site to minimise the range of external influences. Although this might limit the transferability of the findings to other hospital settings, to our knowledge this will be the first study to capture the opinions of hospital surgeons on this issue.

The findings from this research might be used to guide strategy and policy in both clinical and institutional levels around health promotion planning and practice. Gaining the insights from surgeons will be an important step towards the proposed reorientation of hospital practice towards more integrated health promotion settings, culminating in environments that permit patients to take active roles in preventative health and management.

## Additional file

Additional file 1: Surgeons and Preventative Health Survey. (PDF 542 kb)

## Abbreviations

ANZCTR: The Australian New Zealand Clinical Trials Registry
COREQ: Consolidated criteria for reporting qualitative research
