# Supplementary material for: Surgeons and preventative health: protocol for a mixed methods study of current practice, beliefs and attitudes influencing health promotion activities amongst public hospital surgeons
Source: BMC Health Serv Res. 2018 Oct 16;18:780. doi: 10.1186/s12913-018-3606-8 (PMC6192294; doi:10.1186/s12913-018-3606-8)
Supplement: Supplementary file 1 — Surgeons and Preventative Health Survey. (PDF 542 kb) [file 12913_2018_3606_MOESM1_ESM.pdf]

Surgeons and Preventative Health Survey

This survey takes approximately 15-20 minutes to complete.
This survey has been adapted with permission from a survey used by Laws et al, 2008 doi:10.1186/1472-6963-8-44. We would like to thank the authors for their permission to use the survey.

It will be analysed by researchers and reported as group findings- no individual responses will be reported.

Clinician details

Gender: ❑ Female ❑ Male

Age:

Working status: ❑ Full-time ❑ Part-time

If part-time, how many hours do you work per week? _______

Clinician type: ❑ General Surgeon (GS) ❑ Orthopedic Surgeon (OS)
❑ (GS) Registrar ❑ (OS) Registrar

How many years of clinical practice have you undertaken? ____________

Please circle the appropriate answer

Management of risk factors

1. Thinking of the new clients that you have seen over the **past 2 weeks**, what percentage of these clients did you **ask** about the following risk factors:

**Smoking**	None	1-25%	26-50%	51-75%	>75%
**Nutrition**	None	1-25%	26-50%	51-75%	>75%
**Alcohol**	None	1-25%	26-50%	51-75%	>75%
**Physical Activity**	None	1-25%	26-50%	51-75%	>75%

2. Thinking of the clients that you have seen for **review appointments** over the **past 2 weeks,** what percentage of these clients did you **ask** about the following risk factors:

**Smoking**	None	1-25%	26-50%	51-75%	>75%
**Nutrition**	None	1-25%	26-50%	51-75%	>75%
**Alcohol**	None	1-25%	26-50%	51-75%	>75%
**Physical Activity**	None	1-25%	26-50%	51-75%	>75%

3. Of the clients that you identified as having a lifestyle risk factor in the **past 2 weeks**, what percentage of these clients did you **assess their readiness to change their behaviour** (stage of change):

**Smokers**	None	1-25%	26-50%	51-75%	>75%	did not identify any smokers
**Clients with poor nutrition**	None	1-25%	26-50%	51-75%	>75%	did not identify any clients with poor nutrition
**Clients with at risk drinking**	None	1-25%	26-50%	51-75%	>75%	did not identify any clients with at risk drinking
**Physically inactive clients**	None	1-25%	26-50%	51-75%	>75%	did not identify any clients who were physically inactive

4. Of the clients that you identified as having a lifestyle risk factor in the **past 2 weeks**, what percentage of these clients did you provide **verbal advice** to:

**Smokers**	None	1-25%	26-50%	51-75%	>75%	did not identify any smokers
**Clients with poor nutrition**	None	1-25%	26-50%	51-75%	>75%	did not identify any clients with poor nutrition
**Clients with at risk drinking**	None	1-25%	26-50%	51-75%	>75%	did not identify any clients with at risk drinking
**Physically inactive clients**	None	1-25%	26-50%	51-75%	>75%	did not identify any clients who were physically inactive

5. Of the clients that you identified as having a lifestyle risk factor in the **past 2 weeks**, what percentage of these clients did you provide **written advice (e.g. pamphlet, summary sheet etc)** to:

**Smokers**	None	1-25%	26-50%	51-75%	>75%	did not identify any smokers
**Clients with poor nutrition**	None	1-25%	26-50%	51-75%	>75%	did not identify any clients with poor nutrition
**Clients with at risk drinking**	None	1-25%	26-50%	51-75%	>75%	did not identify any clients with at risk drinking
**Physically inactive clients**	None	1-25%	26-50%	51-75%	>75%	did not identify any clients who were physically inactive

6. Of the clients that you identified as having a lifestyle risk factor in the **past 2 weeks**, what percentage of these clients did you **refer to other service providers/agencies or support groups (quitline)** for help in managing their risk factor:

**Smokers**	None	1-25%	26-50%	51-75%	>75%	did not identify any smokers
**Clients with poor nutrition**	None	1-25%	26-50%	51-75%	>75%	did not identify any clients with poor nutrition
**Clients with at risk drinking**	None	1-25%	26-50%	51-75%	>75%	did not identify any clients with at risk drinking
**Physically inactive clients**	None	1-25%	26-50%	51-75%	>75%	did not identify any clients who were physically inactive

7. When you provide advice about lifestyle risk factors, **how much time do you estimate that you spend** on average addressing each of the following:

**Smoking advice**	1-2 mins	3-5 mins	6-10 mins	11-15 mins	More that 15 mins	Do not provide advice
**Nutrition advice**	1-2 mins	3-5 mins	6-10 mins	11-15 mins	More that 15 mins	Do not provide advice
**Alcohol advice**	1-2 mins	3-5 mins	6-10 mins	11-15 mins	More that 15 mins	Do not provide advice
**Physical activity advice**	1-2 mins	3-5 mins	6-10 mins	11-15 mins	More that 15 mins	Do not provide advice

8. For clients that you have given advice to about their lifestyle, what percentage of these clients do you **check their progress** in subsequent visits (on average):

**Smokers**	None	1-25%	26-50%	51-75%	>75%	did not identify any smokers
**Clients with poor nutrition**	None	1-25%	26-50%	51-75%	>75%	did not identify any clients with poor nutrition
**Clients with at risk drinking**	None	1-25%	26-50%	51-75%	>75%	did not identify any clients with at risk drinking
**Physically inactive clients**	None	1-25%	26-50%	51-75%	>75%	did not identify any clients who were physically inactive

9. How easy is it to find **accessible services/agencies/support programs to refer** your clients to for the following?
(1=Very difficult, 5= very easy)

	**Very difficult**			**Very easy**		
**Smoking cessation**	1	2	3	4	5	Don’t know
**Nutrition counselling**	1	2	3	4	5	Don’t know
**Alcohol counselling**	1	2	3	4	5	Don’t know
**Physical activity**	1	2	3	4	5	Don’t know

10. Please rate your **knowledge** in relation to each of the following (1=very poor, 5= excellent)

	**Very poor**				**Excellent**
Assessing nicotine dependency	1	2	3	4	5
**Smoking cessation recommendations**	1	2	3	4	5
**Assessing nutrition**	1	2	3	4	5
**Nutrition recommendations**	1	2	3	4	5
**Assessing for risk alcohol consumption**	1	2	3	4	5
**Recommendations for safe alcohol consumption**	1	2	3	4	5
**Assessing physical activity levels**	1	2	3	4	5
**Physical activity recommendations**	1	2	3	4	5
**Motivational Interviewing**	1	2	3	4	5
**Assessing a client’s readiness to change**	1	2	3	4	5

11. Please rate how **confident** you are in undertaking the following activities with clients
(1=not at all confident, 5= very confident)

	Not at all confident			Very confident	
Assessing nicotine dependency	1	2	3	4	5
Smoking cessation recommendations	1	2	3	4	5
Assessing nutrition	1	2	3	4	5
Nutrition recommendations	1	2	3	4	5
Assessing for risk alcohol consumption	1	2	3	4	5
Recommendations for safe alcohol consumption	1	2	3	4	5
Assessing physical activity levels	1	2	3	4	5
Physical activity recommendations	1	2	3	4	5
Motivational Interviewing	1	2	3	4	5
Assessing a client's readiness to change	1	2	3	4	5

12. Please rate how **effective** you think your advice is in helping clients to:
(1=not at all effective, 5= very effective)

	Not at all effective			Very effective		
**Give up smoking**	1	2	3	4	5	Do not provide advice
**Improve nutrition/ eating habits**	1	2	3	4	5	Do not provide advice
**Reduce alcohol consumption**	1	2	3	4	5	Do not provide advice
**Become more physically active**	1	2	3	4	5	Do not provide advice

13. Clients I see find it agreeable for me to raise the following lifestyle issues routinely as part of consultation:
(1=strongly agree, 5= strongly disagree)

	Not at all effective			Very effective		
**Smoking**	1	2	3	4	5	Do not discuss smoking
**Nutrition**	1	2	3	4	5	Do not discuss nutrition
**Alcohol consumption**	1	2	3	4	5	Do not discuss alcohol
**Physically activity**	1	2	3	4	5	Do not discuss physical activity

14. Please rate **how important** you think the following lifestyle changes are for **improving health**:
(1=not at all important, 5= very important)

	Not at all important				Very important
**Giving up smoking**	1	2	3	4	5
**Improving nutrition/ eating habits**	1	2	3	4	5
**Reducing alcohol consumption**	1	2	3	4	5
**Becoming more physically activity**	1	2	3	4	5

15. Please rate **how important** you think it is to address these lifestyle risk factors with the clients you see:
(1=not at all important, 5= very important)

	Not at all important				Very important
**Smoking**	1	2	3	4	5
**Nutrition**	1	2	3	4	5
**Alcohol consumption**	1	2	3	4	5
**Physically activity**	1	2	3	4	5

16. How much of a **work priority** is it for you/your tem to address lifestyle risk factors with clients as **part of your normal clinical work**:
(1=not at all important, 5= very important)

	Very low priority				Very high priority
**Smoking cessation**	1	2	3	4	5
**Poor nutrition**	1	2	3	4	5
**At rick alcohol consumption**	1	2	3	4	5
**Inadequate physical activity**	1	2	3	4	5

17. In the past 12 months have you had any education or training in the management of these risk factors or strategies towards helping clients change their behaviour?

Smoking	Yes	No
Nutrition	Yes	No
Alcohol	Yes	No
Physical Activity	Yes	No
Motivational Interviewing	Yes	No
Assessing clients readiness to change	Yes	No
Client education	Yes	No

18. Any other comments:____

____

Thank you for completing the questionnaire.

**OFFICE USE ONLY**	**Date received**	**Date entered**	**Entered by (ID)**
